# Operating with Data - Statistics for the Cardiovascular Surgeon: Part I. Fundamentals of Biostatistics

**DOI:** 10.21470/1678-9741-2018-0186

**Published:** 2018

**Authors:** Gabriel Romero Liguori, Luiz Felipe Pinho Moreira

**Affiliations:** 1 Laboratório de Cirurgia Cardiovascular e Fisiopatologia da Circulação (LIM-11), Instituto do Coração (InCor), Hospital das Clínicas HCFMUSP, Faculdade de Medicina, Universidade de São Paulo, São Paulo, SP, Brazil.

The concept of critical reading of research articles is widely accepted as essential for the practice of Medicine. It is known that the use of evidence-based practices can lead to better care and outcomes, thus, it is fundamental that the medical doctor is able to critically appraise the available literature. If the surgeon who reads us aims not only to learn from literature but also to perform research him or herself, it is even more important to be very well informed about the methods involved in scientific studies.

Among the several topics embraced within the scientific method, probably the most discussed and less understood is statistical analysis. For this reason, we created this editorial series entitled "Operating with Data - Statistics for the Cardiovascular Surgeon". The series will merit five editorials, each one describing a different aspect of statistical analysis relevant for the cardiovascular surgeon, as follows:

- Part I. Fundamentals of Biostatistics
- Part II. Association and Risk
- Part III. Comparing Groups
- Part IV. Correlations and Regression
- Part V. Survival Analysis

In this first editorial, we will address the fundamental concepts required for understanding Biostatistics.

## Types of Variables in Statistics and Research

An important initial concept everyone should understand is that "data", as a term originated from the Latin language, is the plural form of "*datum*", which, in turn, meant "something given". So, "*datum*" is the minimal amount of information one can describe. This "*datum*" could be translated as a single value of a determined variable (*e.g*. a single measurement of the blood glucose) and it can assume different interrelation with other "*datum*", as well as diverse ways to be described. In this regard, according to how the "*datum*" behaves, the variable represented by it can be classified in different manners.

The first manner to classify a variable is describing it as independent or dependent. Independent variables are those which their data are not modified by any other data. An example of an independent variable is the study group. Once determined by the researcher, the study group cannot be modified by any other data. The opposite of an independent variable, naturally, is a dependent variable, described as the one that can be modified by other variables - in this case, the independent variables. An example of dependent variable could be the mortality rate of a treatment. The mortality rate does not modify other parameters but is modified by, for instance, the study group. So, in a scenario in which the researcher is willing to understand if three different surgical techniques - A, B, and C - result in different mortality rates, the study group *i.e*. the surgical technique is the independent variable and the mortality rate is the dependent one. A variable can assume both independent or dependent behavior, but they can never coexist in the same analysis. Let's say we use blood glucose as a variable. If we are willing to understand how blood glucose influence, for instance, the infection rates after cardiovascular procedures, blood glucose is an independent variable. On the other hand, if we want to evaluate the effects of different diets on blood glucose, blood glucose behaves as a dependent variable.

**Fig. 1:**
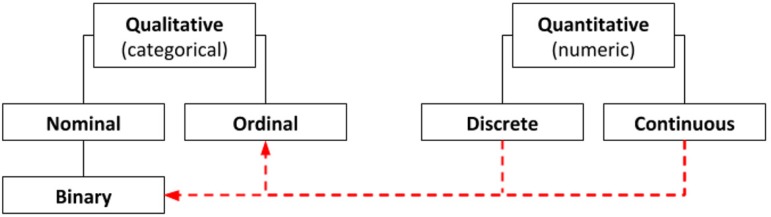
Types of variables in statistics and research. For practical purposes, quantitative variables can be converted into qualitative variables.

The second manner to classify a variable is according to how it can be described. This classification includes two main groups, four subgroups, and one subtype (Figure 1). The first group belongs to the qualitative variables. These variables are those which are not numeric, but represent categories; for this reason, they can also be called categorical variables. Qualitative variables can be subdivided into two subgroups, the nominal and the ordinal variables. Nominal variables are those in which categories do not present a natural order (*e.g.* blood type). Nominal variables present a particular subtype denominated binary variables; these variables are those in which there are only two possible and opposite categories, like yes/no, present/absent, live/dead. Ordinal variables, in turn, are those in which a natural order exists within the categories [*e.g.* New York Heart Association (NYHA) classification of heart failure]. For these variables, it is possible to determine which categories come before and after each other. The second group of variables is represented by the quantitative variables. Quantitative variables embrace data that can be objectively measured and represented by numbers, like dimensions, concentrations, time, etc.; for this reason, they can also be called numeric variables. These variables can also be divided into two subgroups, the discrete and the continuous variables. Discrete variables are defined as those which data can only be represented by integers (*e.g.* number of surgeries). Continuous variables, however, allow data to be expressed as any number within the set of real numbers, including numbers with decimal places (*e.g.* hemoglobin count). For practical purposes, quantitative variables can be converted into qualitative variables, more specifically into ordinal or binary variables. Let's say, for instance, that we have a dataset with the age of a series of patients. We could use these numeric values as they are, but, maybe, for a particular study, what really matters is a potential difference between children, adult, and elderly patients. Thus, we could convert the numeric values of age into categorical values, separating them into three groups: children (0-18 years), adult (>18-65 years), and elderly (>65 years); this case represents the conversion of a discrete variable into an ordinal variable. Another interesting example can be described by the categorization of blood glucose levels. Here, instead of using the exact value given by the blood glucose test, we could separate them into two categories: non-diabetic (<126 mg/dL) and diabetic (≥126 mg/dL); this case represents the conversion of a continuous variable into a binary variable.

## The Ways Quantitative Data Can Be Distributed

**Fig. 2:**
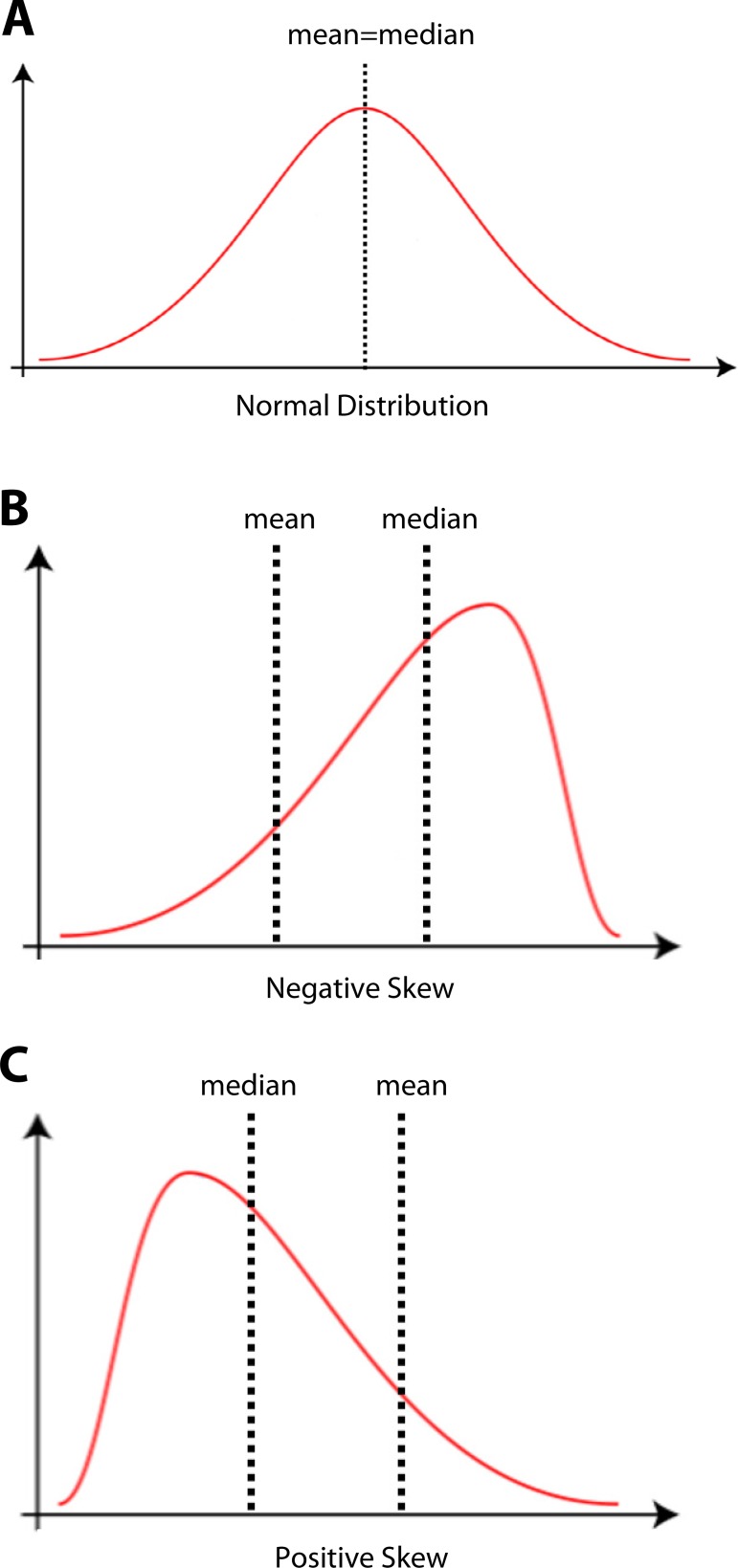
The ways quantitative data can be distributed. A. Normal distribution. B. Negative-skewed distribution. C. Positive-skewed distribution.

Quantitative variables can be described according to the distribution of their data. This distribution can be better understood if we consider an XY graph in which the X-axis represents the different values a variable can assume, and the Y-axis represents the number (or percentage) of times the values in X-axis appear within the whole sample. Although data can be distributed in virtually any manner, there are two most common ways for it to happen. The first is what is called normal distribution, also known as symmetric or Gaussian distribution (Figure 2A). In this kind of distribution, the representation of the frequency of each value in the sample results in a bell-shaped curve in the XY graph explained above, with the peak of the curve representing the mean and the median - concepts which will be explained later in this editorial - simultaneously and equal distributions being found to the left and right sides of this peak, as mirror images. The normal distribution is the rule for most of the biological variables. The second type of data distribution is the skewed distribution, also referred to as asymmetrical distribution. This kind of distribution is found when data is clustered toward one end of the distribution curve and there is no value in the X-axis that can divide the curve into two equal parts. Indeed, in the skewed distribution, the mean and the median of the data do not coincide and none of them are at the peak of the curve. The skewed distribution can be found in two different forms, the negative-skewed distribution (or left-skewed distribution) and the positive-skewed distribution (or right-skewed distribution). In the negative-skewed distribution, data is clustered in the right side of the graph and the skew *i.e.* the long tail is to the left, resulting in mean and median being moved to the left side of the peak, with the mean being left to the median (Figure 2B). Exactly the opposite is found in the positive-skewed distribution: data is clustered in the left side of the graph and the skew is to the right, resulting in mean and median being moved to the right side of the peak, with the mean being right to the median (Figure 2C). Although exceptions exist, and some rare skewed distributions can show the mean in the opposite side of what is expected^[^^1^^]^, for practical purposes the rules described above are valid for virtually any variable in medical research. Although most variables can be considered as normal distributed, several situations may lead to a skewed distribution of biological variables, thus every dataset must be analyzed individually. To reveal if your data is normally distributed or not, it is possible to use specific statistical tests. The two most commonly used are the Shapiro-Wilk test, indicated to samples smaller than 50 subjects, and the Kolmogorov-Smirnov test, for samples greater than 50 subjects.

## How to Present your Data

Once one knows what the kind of variables are and what is the distribution of the data that will be analyzed, it becomes easy to know how this data must be presented.

### Qualitative Variables

Qualitative variables are generally described as the proportion - usually the percentage - of subjects within each category of the sample. This simple calculation will result in a single value (*e.g.* 50%); however, it is possible to determine a confidence interval for that value using the sample size (n) and the critical value of the standard normal distribution. The concepts of confidence interval and critical value of the standard normal distribution will be discussed in the next section of this editorial, but for now it is only necessary to know that the confidence interval is a range of values in which there is a specified probability that the value of the studied variable lies within and that the critical value of the standard normal distribution for a 95% confidence interval (CI 95) is 1.96. Thus, the CI 95 of a proportion (p) can be defined by the following formula:

$$\mathrm{CI}\;95=p\pm 1.96\sqrt{\frac{p\left(1-p\right)}{n}}$$

The graphical representation of qualitative variables can be done with several different graphs that allow showing proportion, but two of them are the most commonly used. The first of them is the pie chart, in which proportions are displayed as "slices of a pie". This kind of graph should be saved for variables containing few categories and large sample sizes, otherwise they can become confusing and/or misleading. The second type of graph commonly used to represent proportions is the bar chart, in which the values for each category are represented by the height or length of bars with equal width. The bar chart can be combined with the confidence interval of the proportion (as described above) to constitute a more robust graphical representation.

### Quantitative Variables

Quantitative variables are mathematically represented by a measure of central tendency followed by a measure of statistical dispersion.

Measures of central tendency are values that can represent the data within a single number. The most commonly used measures of central tendency in medical research are the arithmetic mean, or simply mean, and the median. The mean is the sum of the values of all observations divided by the number of observations. The median, in turn, is the value separating the higher half of a sample from the lower half, *i.e*. the value at the exact middle of the set of values. When the sample is composed of an even number of observations, the median is the mean of the two central values. Mean and median present, both, advantages and disadvantages. While mean allows broad possibilities of algebraic treatment (and consequently statistical analysis), it is affected by extreme values *i.e*. outliers that can distort results. The median, on the other hand, is a more robust measure, not being affected by aberration values, and is also an easy to understand measure - 50% of the sample is below it and 50% is above it. However, median does no account for all observations and, thus, presents limited possibilities for algebraic treatment.

**Fig. 3:**
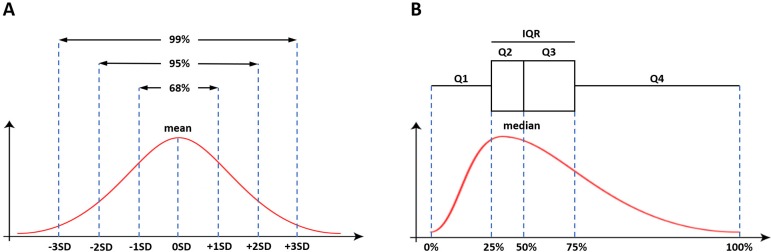
The measures of central tendency and dispersion. A. Mean and standard deviation (SD) in a normally distributed sample. B. Median and interquartile range (IQR) in a non-normally distributed sample.

The measures of central tendency are always followed by a measure of statistical dispersion, which, in turn, represent how the sample is distributed around the measure of central tendency. The sample could be, for instance, clustered around the mean or median, but could also be spread far from them. The two most important measures of statistical dispersion for medical research are the standard deviation (SD) and interquartile range (IQR). SD is mathematically defined as the square root of the variance of a given dataset. For practical purposes, however, what is important to understand is that the value of the SD represents a range of values in which a determined percentage of the sample lies within it, in a normally distributed dataset. For this reason, SD is always used together with the mean - not the median. The rule is that, if you take the values lying between -1SD and +1SD from the mean, you will have 68% of the sample; if you take between -2SD and +2SD, you will have 95% of the sample; and if you take between -3SD and +3SD, you will have 99% of the sample (Figure 3A). This is the reason why a 95% CI is defined within the 2SD (in fact, 1.96SD) limits; these limits are the 95% critical value of the standard normal distribution (this is the origin of the formula to calculate the CI 95 of a proportion, as stated previously). For skewed curves, however, the mean and SD cannot infer the same patterns as those found in the normal distributions and, thus, the median and IQR are necessarily used. The IQR is calculated as the difference between the 75^th^ and the 25^th^ percentiles. Differently from SD, IQR cannot give more information than what is already known to calculate it *i.e*. the range of values that embrace 50% of the observations in the middle of the sample. IQR can be used, however, to construct the box plot charts employed for the representation of non-normal distributions (Figure 3B).

**Fig. 4:**
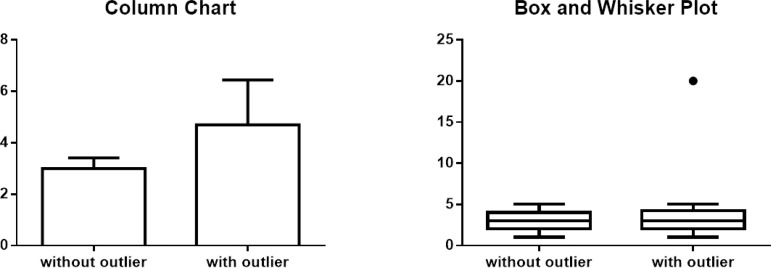
Graphical representation of quantitative variables. The same datasets are represented with bar chart and box and whisker plot. While column chart is strongly affected by an outlier, the box and whisker plot remains essentially the same.

The graphical representation of quantitative variables can be done in several ways, but it will majorly depend on the distribution of the data. For normally distributed samples, the most commonly used graph is the column chart with error bars in which the top of the column is the mean and the error bars represent the SD or standard error of the mean (SEM), which is the SD divided by the square root of the sample size (the lower the SEM, the more representative the sample). While SD describes the dispersion of the measured values, SEM represents the range within there is a probability of 68% to include the real value of the mean of the study population. For non-normal distributions, in turn, the most frequently chosen method of representation is the box and whisker plot. This chart represents 1) the median, 2) the IQR and 3) either the total range of the values (minimum and maximum) or the 1.5 IQR range with outliers. The use of 1.5 IQR range with outliers was proposed by the mathematician John W. Tukey^[^^2^^]^, in the 1970's and, since then, has been widely used for the representation of non-normal distributions; still, the use of 1.5 IQR as the limit for outliers was chosen by convenience. Box and whisker plots can also be used for normally distributed data, although the opposite - to use column charts with error bars for non-normally distributed data - is not appropriate (Figure 4). Besides these two graphical representation methods, many other exists both for normal and non-normal distributions, but the ones explained in this editorial are the most used.

## (In)famous Statistical Entities in Medical Research

Probably the most famous entity in medical research statistics is the *P*-value. But what does it mean? The *P*-value is a value ranging from 0 to 1 *i.e*. 0% to 100% that will tell what is the probability that a difference (or an association, a correlation, etc.) was found due to chance. This - finding a difference when it does not exist - is called type I error. Usually, in the medical literature, we can find the use of a predefined threshold for *P*-value named level of significance (α). Most of the time, this level of significance is arbitrary defined as 0.05 (or 5%) and is set as the limit above which the result of a test cannot be considered as true. However, in many situations, we will find *P*-values considered to be marginal *i.e*. they are close to the 0.05 cutoff. For this reason, we - and the reviewers and editors from other journals^[^^3^^,^^4^^]^ - advocate that authors always report the exact *P*-value so that the readers can draw their own conclusions. Some people might accept a 10% probability of error, while others might prefer to be right 99% of the time. An alternative to the use of *P*-values is the use of CI. Ideally, both should be reported together. The CI limits a range in which there is a determined percentage of certainty that the true value of the population lies within; in the medical literature, it is most commonly used the 95% CI, that coincides with the 5% level of significance. It is important to notice that the CI is not the range that contains a determined percentage of the values.

Another frequently discussed entity in medical research statistics is the sample size. It is easily understandable that small samples present a limited capacity for estimating the findings of the real population, as well as that large samples have a high precision to perform these estimations. But what is the minimum sample size required to find results which can be extrapolated to the real population? To calculate sample size, a series of factors must be observed in advance. First, what is the minimum difference considered to be clinically relevant? Second, what is the pattern of dispersion of this variable - how broad is the SD/IQR? Third, what are the expected losses in follow-up? Fourth, what is the statistical test to be performed for analyzing differences for this variable? And fifth, what are the limits for type I and type II errors we are willing to accept. Type I error was defined above. The type II error is the opposite *i.e*. not finding a difference when it does exist. While type I error is defined as α *i.e*. the level of significance, type II error is defined as β. Calculating 1-β you will find what is defined as the power of the test *i.e*. the probability of correctly inferring a difference. A level of confidence of 5% (α=0.05) and a power of 80-90% (0.1<β<0.2) are the standard values used for calculating sample size. The exact calculation of the sample size, as explained above, will depend on the statistical tested to be used. For this reason, further detailed discussion on sample size calculation for each kind of test will be carried in the next editorials.

## A Few Concepts to Choose the Right Statistical Test

After understanding the types of variables, the ways data can be distributed and how we can present our findings, it is fundamental, now, to learn about statistical tests - how they work and when to choose each of them. Before that, however, and to close this editorial on the fundamentals of Biostatistics, we will present a few concepts necessary for choosing the right statistical tests.

The first fundamental concept in this regard is the difference between parametric and non-parametric tests. Parametric tests are the ones that assume specific parameters *i.e*. distributions for the sample. Parametric tests assume that the sample distribution is normal, and all the calculations are based on this assumption. Some famous examples of parametric tests are the t-tests, the analysis of variance (ANOVA), and the Pearson coefficient of correlation. Non-parametric tests, in turn, are those that do not assume any specific distribution of the sample. These tests use other means (*e.g*. ranking) to calculate probabilities. Besides non-normal distributions, non-parametric tests are also indicated for small sample sizes. Examples of non-parametric tests are Wilcoxon tests, the Kruskal-Wallis test, and the Spearman's rank correlation. Since parametric tests are based on normal distributions, they use the mean as a measure of central tendency, while non-parametric tests use the median. In conclusion, parametric tests should be used for normally distributed samples and non-parametric tests for non-normally distributed samples. Still, the use of non-parametric tests for normally distributed samples is possible, but the opposite - using parametric tests for non-normally distributed samples - is not appropriate.

The second concept which is important to understand when choosing a statistical test is the difference between paired and unpaired data. Paired data are data which are somehow related. Usually, paired data refers to measurements taken before and after a given procedure (*e.g.* measuring transvalvular pressure gradient before and after valvuloplasty). However, data can be considered paired also in other situations, for instance when comparing an intervention in a specific anatomic structure with its contralateral equivalent [*e.g*. implanting an experimental tissue-engineered blood vessel in the left carotid artery and a control polytetrafluoroethylene (PTFE) tube in the right carotid artery of the same animal]. Other more extreme examples of paired data are studies using twins, husband/wives, brothers/sisters, and matched cases. Still, properly matching cases - for example by sex, age, body mass index (BMI), etc - is difficult and perfect matches rarely can be achieved. Unpaired data, in turn, are those which there is no link between the subjects submitted to the measurement of the variable. The importance to define if a dataset is composed of paired or unpaired data is because paired tests are considered more powerful to identify differences related to the intervention. In these cases, the variability of the sample is bypassed by using each subject is his/her own control.

Understanding when data is normal or non-normal distributed *i.e*. requires parametric or non-parametric tests, and if data is paired or unpaired is the basic requirement to know which statistical test to choose. The detailed discussion on which test to use for each type of research question will come in the next editorials.
